# Genome-wide diversity and structure variation among lablab [*Lablab purpureus* (L.) Sweet] accessions and their implication in a Forage breeding program

**DOI:** 10.1007/s10722-021-01171-y

**Published:** 2021-03-19

**Authors:** Julius Pyton Sserumaga, Siraj Ismail Kayondo, Abasi Kigozi, Muhammad Kiggundu, Clementine Namazzi, Kato Walusimbi, James Bugeza, Allen Molly, Swidiq Mugerwa

**Affiliations:** 1 National Agricultural Research Organization; National Livestock Resources Research Institute, P.O. Box 5407, Kampala, Uganda; 2 Present Address: International Institute of Tropical Agriculture, Ibadan, Nigeria

**Keywords:** Plant genetic resources, Genetic differentiation, Genetic diversity, SNP, SilicoDart

## Abstract

Most orphan crops have not been fully sequenced, hence we rely on genome sequences of related species to align markers to different chromosomes. This hinders their utilisation in plant population improvement programs. Utilising the advances in the science of sequencing technologies, the population structure, relatedness, and genetic diversity among accessions can be assessed quickly for better exploitation in forage breeding programs. Using DArTseq technology, we studied the genetic and structural variation in 65 *Lablab purpureus* (L.) Sweet conserved gene-bank accessions using 9320 DArTseq-based SNPs and 15,719 SilicoDart markers. These markers had a low discriminating ability with mean polymorphic information content (P.I.C.) of 0.14 with DArTseq-based SNPs and 0.13 with SilicoDart markers. However, the markers had a high mean call rate of 73% with DArTseq-based SNPs and 97% with SilicoDart markers. Analysis of molecular variance revealed a high within populations variance (99.4%), indicating a high gene exchange or low genetic differentiation (PhiPT = 0.0057) among the populations. Structure analysis showed three allelic pools in variable clusters of ΔK = 3 and 6. Phylogenetic tree of lablab accessions showed three main groups with variable membership coefficients. Most pairs of accessions (40.3%) had genetic distances between 0.10 and 0.15 for SilicoDart markers, while for DArTseq-based SNPs, (46.5%) had genetic distances between 0.20 and 0.25. Phylogenetic clustering and minimum spanning analysis divided the 65 accessions into three groups, irrespective of their origin. For the first time, this study produced high-density markers with good genom coverage. The utilisation of these accessions in a forage program will base on the information from molecular-based grouping. The outcomes uncovered the presence of noteworthy measure of variety in Uganda, CIAT and ILRI accessions, thus demonstrating an opportunity for further marker-trait-association studies.

**Supplementary Information:**

The online version contains supplementary material available at 10.1007/s10722-021-01171-y.

## Introduction

Dolichos lablab [*Lablab purpureus* (L.) Sweet] is an essential legume used as food and feed. It is assumed to have originated in Africa (Maass et al. 2005; Maass and Usongo 2007; Verdcourt 1970) and India (Ayyangar and Nambiar 1935; Shivashankar et al. 1993). It belongs to the family Fabaceae characterised as a busy semi-erect perennial herb. It is primarily a self-pollinated crop with doubled chromosome number 2n = 2x = 22 (Goldblatt 1981; She and Jiang 2015). It is one of the diverse annual legume crop in tropical and subtropical regions worldwide (Smýkal et al. 2015). Lablab is a multipurpose crop used mainly for animal feeding as forage meal, fresh forage, straw, hay, grain, grazing, or browsing. Humans consume it’s fresh leaves, immature grains, mature grains, green pods, as pharmaceutical or nutraceutical foods (Adebisi and Bosch 2004; Morris 2009; Subagio and Morita 2008). The crop is also used for soil improvement, protection and weed control (Ewansiha and Singh 2006).

In Uganda, lablab is predominantly utilised as feed for ruminants, notably cattle, served as fresh foliage. The use of lablab grain in monogastric and ruminant diets is limited due to high levels of antinutritional compounds in locally available cultivars. Furthermore, the utilisation of lablab for silage production is constrained by the incompatibility of available local cultivars with silage production equipment and difficulties in wilting the crop due to its thick moist stems. The crop’s intolerance to trampling and grazing also constrains the integration of lablab into grass-dominated pasture swards under grazing systems. Regardless of its wide adaptability, diversity and aptness to tropical agricultural production systems, lablab remains underutilised (Ebert 2014; Engle and Altoveros 1999). In the effort to harness the multiple benefits of lablab and to stimulate its increased utilisation in diverse livestock production and feeding systems, the National Livestock Resources Research Institute of the National Agricultural Research Organisation of Uganda acquired lablab germplasm from the International Livestock Research Institute (ILRI), the International Centre for Tropical Agriculture (CIAT), local country collections, and assembled a group of elite accessions. This collection of elite accessions is well-thought-out as the most reliable and efficient source for the primary search of trait-specific accessions. These can be utilised for quantitative trait loci discovery, allele mining, and association mapping panel development to explore forward breeding while enhancing the genetic gains in lablab breeding for yield and its component traits.

With recent genomic technological advancement, it’s now possible to examine the whole species’ genome than selected regions within the genome to capture markers that contribute to complex traits (Maulana et al. 2019). Thus, it’s imperative to understand the genetic relationship of new and uncharacterised accessions to effectively be utilised in the breeding pipeline (Sserumaga et al. 2019, 2014). This is possible with the help of molecular markers since their cost per data point is low, highly abundant within the genome, they are locus-specific, co-dominant, and low genotyping error rates (Rafalski 2002). Single nucleotide polymorphism (SNP) are one of the robust marker types used in diversity studies and genome-related association studies (Azmach et al. 2013; Farfan et al. 2015; Suwarno et al. 2015). However, some orphan crops like lablab have not been sequenced to the fullest. This study aimed to determine (i) the level of molecular diversity and structure among 65 gene-bank accessions using 9320 DArTseq-based SNP markers and 15,719 DArTseq-based SilicoDArT markers, (ii) the relationship among the set of accessions for better utilisation in a breeding program.

## Materials and methods

### Plant materials, DNA extraction and Genotyping using DArTseq platform

A total of 65 lablab gene-bank accessions acquired from ILRI and CIAT gene banks and local collections were used in the study (Table 1). Leaf tissue was collected, packaged and shipped for genotyping at Integrated Genotyping Sequence Support (IGSS) platform hosted at Bioscience for East and Central Africa (BecA)—Hub, at ILRI, Nairobi. The leaf samples were lyophilised and total DNA extracted using the DNeasy plant mini kit (250) (Qiagen Inc., Valencia, CA) as per the manufacturer’s guidelines. DNA concentration and purity were determined using a Nanodrop (DeNovix DS-11 FX spectrophotometer). Extra quality check was carried out on 0.8% agarose gel electrophoresis with lambda DNA of 50 ng as a marker. DNA for each sample was diluted to a required concentration range of 50–100 ng/µl for the DArTseq genotyping platform. After standardisation, 25 µl was aliquoted into 96 well semi-skirted plates for sequencing.

**Table 1:** Proportion of membership of each predefined population from structure analysis (ΔK = 3)

Population	Number of Individual	Estimated membership coefficient		
		CI	CII	CIII
CIAT gene banks (CIAT)	39	0.363 (14)	0.106 (4)	0.531 (21)
ILRI gene banks (ILRI)	19	0.313 (6)	0.492 (9)	0.196 (4)
Local Collection (UG)	7	0.035 (0)	0.208 (2)	0.757 (5)

Using DArTseq platform, lablab genotyping was carried out using Diversity Array Technology (http://www.diversityarrays.com/) (Kilian et al. 2012). Digestion of 50 ng of DNA was done using a recipe of *PstI/HpaII* restriction endonucleases. Products later ligated to a *PstI* overhang compatible with oligonucleotide adapter and sequenced using *PstI* site-specific primers on an Illumina HiSeq 2500 (Illumina). Referencing the DArTseq protocol, Short sequence fragments, SNP and SilicoDArT, markers were generated. Since there is no available full sequence of lablab bean, the sequence fragments were aligned with the Mung bean (*Vigna radiata* (L.) R. Wilczek) reference sequence on Ensembl (https://plants.ensembl.org/Vigna_radiata/Info/Index). The genome-wide SNP-density plot distribution of the markers was envisaged using the R-package CMplot (https://github.com/YinLiLin/R-CMplot).

### Marker data analysis

Genotyping by Sequence data for SNP and SilicoDArt markers distributed across the lablab genome was received from IGSS at BecA Hub. Percentage of missing data per marker, per accession, Call rates, polymorphic information content (P.I.C.) and Expected heterozygosity (He) were calculated in DartView (http://software.kddart.com/kdxplore/dartview/). Using TASSEL v.5.2.43 software (Bradbury et al. 2007), genotypic data was filtered with 0.05 for minor allele frequency and a minimum count of 80% for sample size. Genetic distance was computed between pair of lablab accession using identity by state similarity (I.B.S.) method in TASSELv.5.2.43. A marker based kinship matrix was then calculated between pair of lablab in dataset using TASSELv.5.2.43.

### Genetic relationship and population structure

The Diversity of the lablab accessions were assessed using the model-based STRUCTURE, minimum spanning network and different diversity indices Stoddart and Taylor’s G (Stoddart and Taylor 1988) and Shannon–Wiener’s *H’* (Shannon and Weaver 1949). A multivariate model-based clustering approach, implemented in the STRUCTURE software package version 2.3.4 (Pritchard et al. 2000), was used to analyse population structure. In the model-based clustering approach, a 100,000 burn-in period was utilised, followed by 100,000 iterations. A model taking into consideration admixture and correlated allele frequencies with no information about location or population was used to deduce the right number of groups in the population of 65 accessions using posterior probabilities (qK). Ten independent runs of STRUCTURE were executed with the number of clusters set from 1 to 10, through 10 replicates for each K. Delta K was computed for each value of K using online software, the Structure Harvester (Evanno et al. 2005). Each accession was allocated to a given group when the extent of its genome in the cluster (qK) was higher than an edge estimation of 50%.

Phylogenetic analysis using unweighted pair-group mean arithmetic was performed to envisage the relationships between accessions using the R package Analyses of Phylogenetics and Evolution (ape) (Paradis et al. 2004). Analysis of molecular variance (AMOVA) was performed to determine the variance among populations and among accessions within populations using the R package poppr version 2.8.5. (Kamvar et al. 2015). Genetic differentiation among lablab accession populations was calculated with the R packages poppr version 2.8.5 and vegan version 2.0.7 (Kamvar et al. 2015; Oksanen et al. 2013), which enabled the estimation of standardised PhiPT and the allelic patterns across different populations (Meirmans 2006). An independent analysis called the minimum spanning network was used to visualise the population structure using igraph R package version 1.2.5. (Csardi and Nepusz 2006).

## Results

Genotyping lablab accessions using Genotyping by Sequencing.

### Maker Density, genetic distance and relationships

**Fig. 1:**
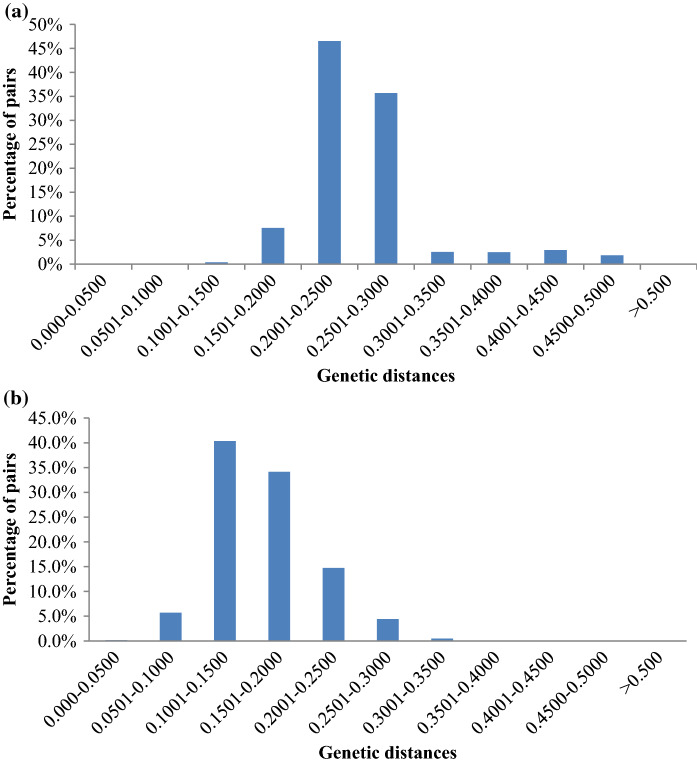
**a** Roger’s genetic distance distribution for 65 Lablab Accessions genotyped with 9320 polymorphic SNPs markers. **b** Roger’s genetic distance distribution for 65 Lablab Accessions genotyped with 15,719 polymorphic SilicoDArT markers

A total of 9,320 polymorphic SNPs makers were called on 65 lablab accessions with an average of two alleles detected per loci and with a mean call rate of 73%. Average minor allele frequency calculated ranged from 0 to 0.09 with a mean of 0.09. Heterozygosity per marker ranged from 0 to 0.61, with a mean of 0.03. Polymorphic Information Content ranged from 0.02 to 0.5, with an average of 0.14. Genetic distance between lablab accession pairs ranged from 0.08 to 0.49, with an average of 0.26. The majority of lablab pairs (46.5%) had genetic distances between 0.20 and 0.25 (Fig. 1a). Relative kinship relationship coefficients between sets of accessions ranged from 0 to 3.85, with an average of 4.42. The genetic differentiation among the ecotype populations (PhiPT) was low (0.0056) (Table 2).

**Table 2:** Genotypic richness, diversity, and evenness

Pop	N	MLG	eMLG	SE	H	G	lambda	E.5	Hexp	Ia	rbarD
CIAT	39	39	10	0.00E+00	3.66	39	0.974	1	0.313	127.6	0.00922
ILRI	19	19	10	2.51E−07	2.94	19	0.947	1	0.304	926.7	0.05827
UGA	7	7	7	0.00E+00	1.95	7	0.857	1	0.363	90.3	0.00913
Total	65	65	10	6.30E−06	4.17	65	0.985	1	0.255	362.6	0.02208

*Pop* Population name, *N* number of individuals observed, *MLG* number of multilocus genotypes (MLG) observed, *eMLG* the number of expected MLG at the smallest sample size ≥ 10 based on rarefaction, *SE* standard error based on eMLG, *H* Shannon–Wiener index of MLG diversity, *G* Stoddart and Taylor’s index of MLG diversity, *lambda* Simpson’s Index, *E.5* evenness, *Hexp* Nei’s unbiased gene diversity, *Ia* the index of association, *rbarD* the standardized index of association

A total of 15,719 SilicoDArT markers were called on the 65 lablab accessions, with a mean call rate of 97%. Polymorphic Information Content ranged from 0.03 to 0.50, with an average of 0.13. Genetic distance between pairs of accessions ranged from 0.03 to 0.32, with a mean of 0.16. The majority of pairs of accessions (40.3%) had genetic distances between 0.10 and 0.15 (Fig. 1b). The relative kinship relationship coefficient between sets of accessions ranged from 0 to 3.25, with a mean of 3.42.

### Genome-wide SNP-density distribution plot of the markers

**Fig. 2:**
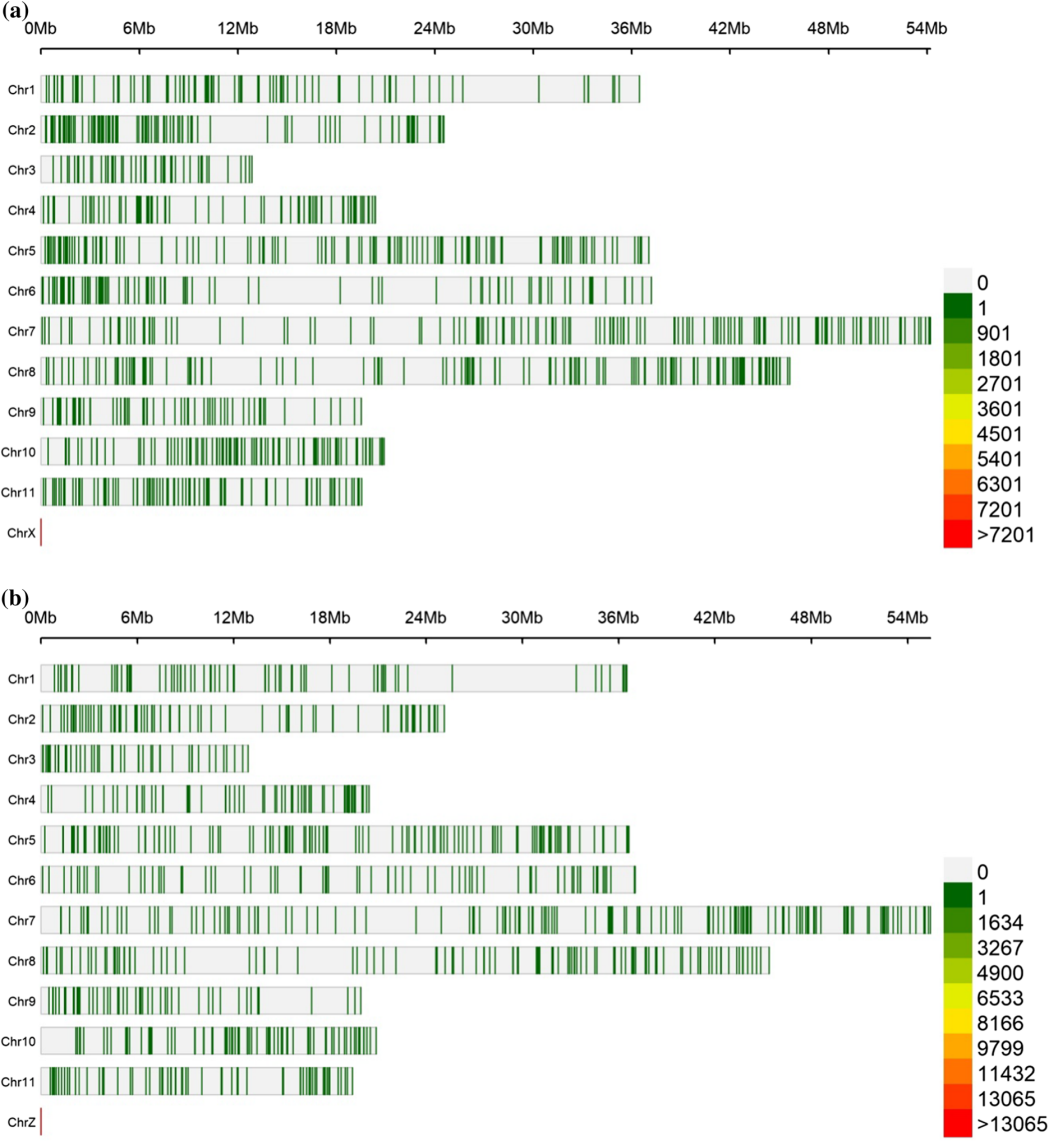
**a** SNP density levels within 1 Mb window size with different colors. “Chr” refers to common mung bean chromosomes with unmapped markers. **b** SilicoDArT Marker density levels within 1 Mb window size with different colors. “Chz” refers to common mung bean chromosomes with unmapped markers

The SNP and SilicoDArT markers were mapped to the genome of Mungbean, because it’s the specie with a sequenced genome closely related to lablab. The markers aligned per chromosome ranged from 49 to 162 for SilicoDArT, and 54 to 167 for SNPs. In both sets of markers, the largest and least number of markers mapped onto chromosome seven and three respectively. Generally, only 7% (1025 out of 15,719) of the SilicoDArT markers and 13% (1226 out of 9320) of the SNP markers mapped on the eleven chromosomes of the Mung bean genome (Fig. 2a, b).

### Phylogenetic analysis

**Fig. 3:**
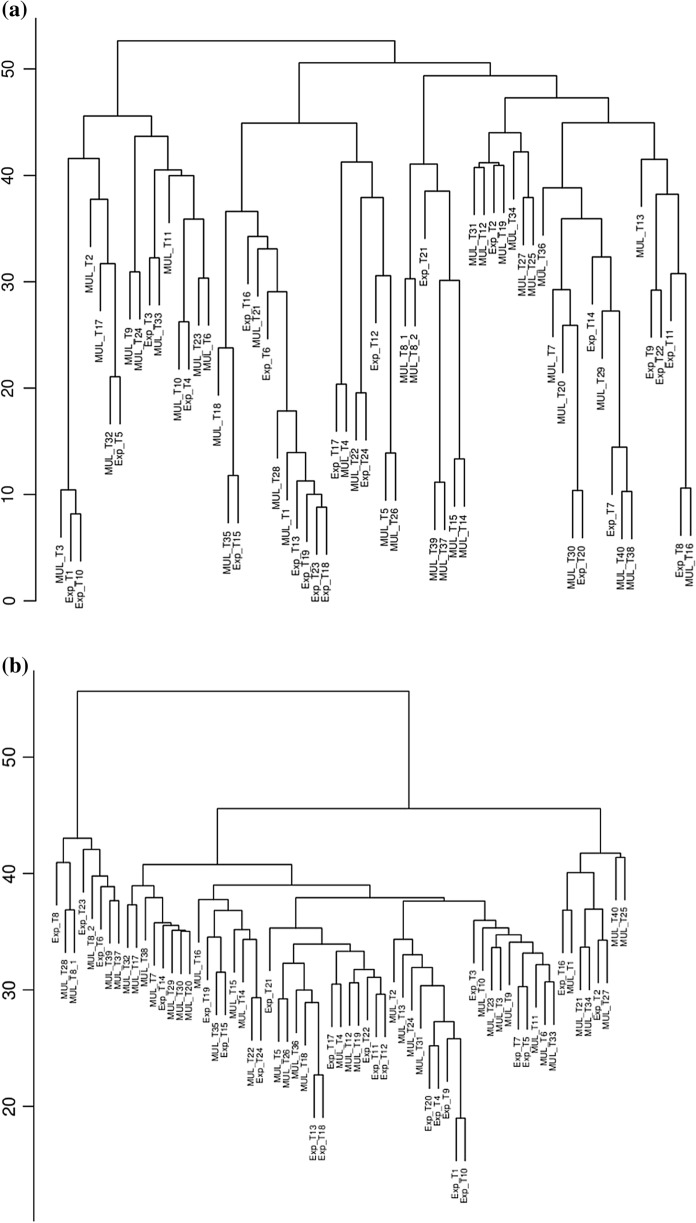
**a** Phylogenetic tree for 65 Accessions dependent on Rogers’ genetic distance from 9320 SNP markers. **b** Phylogenetic tree for 65 Accessions dependent on Rogers’ genetic distance from 15,719 polymorphic SilicoDArT markers

The lablab accessions clustered into three groups at 40–50 distances (Fig. 3a, b). Phylogenetic trees clustered the accessions into three subgroups (Fig. 3a, b). Results from SNP clustering revealed that Group III (46%) consisted of more accessions than Group I (25%) and Group II (29%). SilicoDArT markers clustering revealed that there were more accessions in Group II (75.4%) than in either Group 1 (12.3%) or III (12.3%). Group 1 consisted more of ILRI and CIAT accessions under SNP clustering and only, Ugandan accessions were clustered in Group III. However, using SilicoDArT markers, the Uganda accessions were evenly distributed in all the 3 groups.

SNP clustering established that one ILRI accession was closely related to 15 CIAT accessions in group 1. In group 2, 6 CIAT and 5 ILRI accessions sub grouped with one Ugandan accession. At the same time, the second sub-group comprised only CIAT accessions. In Group 3, 14 CIAT accessions sub grouped with 9 ILRI and 6 Ugandan accessions. Silico Dart marker grouping is more less like SNP clustering. The dendograms (Fig. 3a, b) indicate 3 lineages in the lablab population and similar pattern is observed in clusters generated by STRUCTURE.

**Fig. 4:**
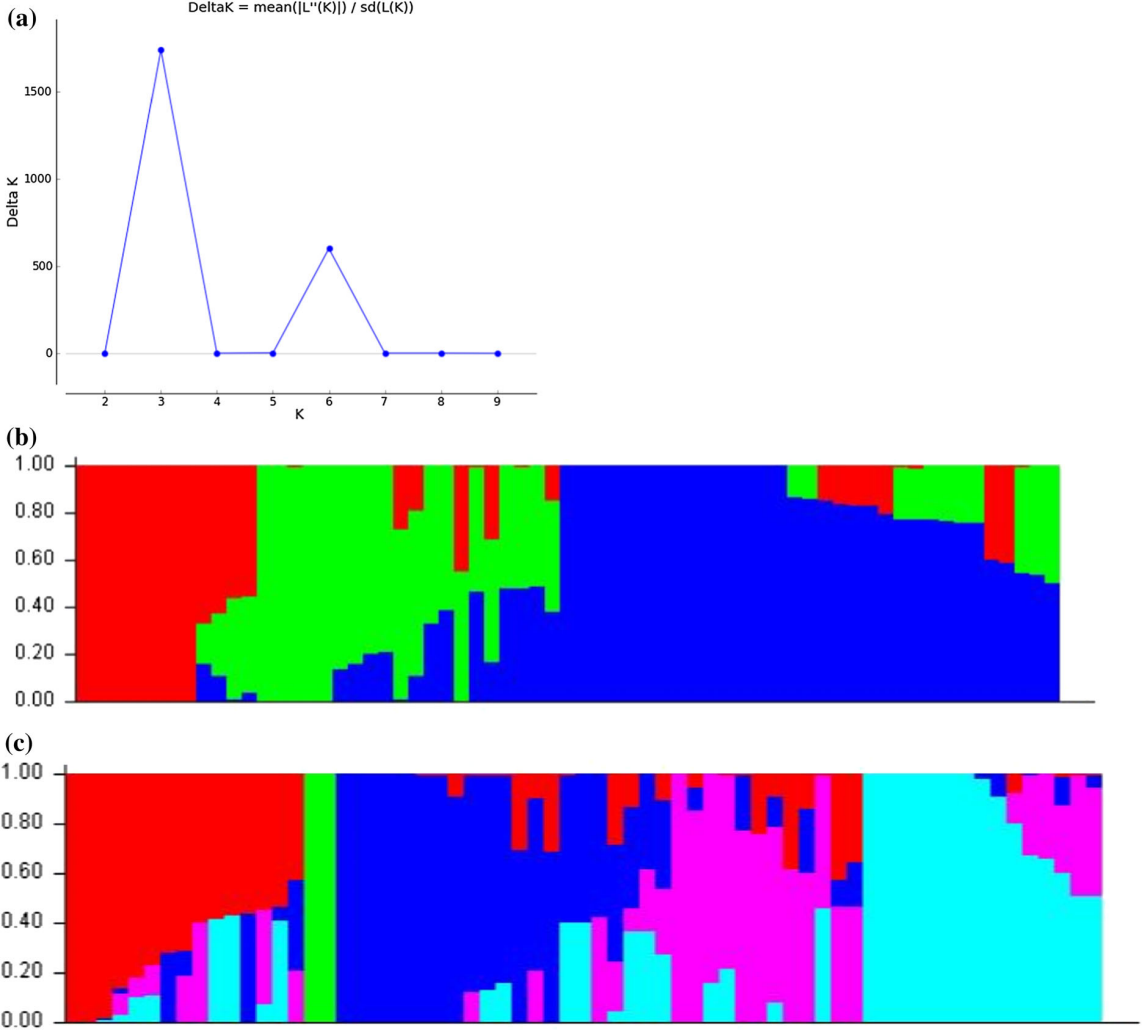
Minimum spanning networks (MSN) of 65 accessions based on origin

Using SNPs for minimum spanning network clustering, the number of clusters detected was also 3, but not based on their origin of the accessions (Fig. 4). The Ugandan accessions was found in two groups. Also, the network (Fig. 4) indicates the presence of 3 lineages in the lablab population, and a similar pattern is observed in clusters generated by STRUCTURE and Neighbor-Joining.

### Diversity in the lablab populations

**Fig. 5:**
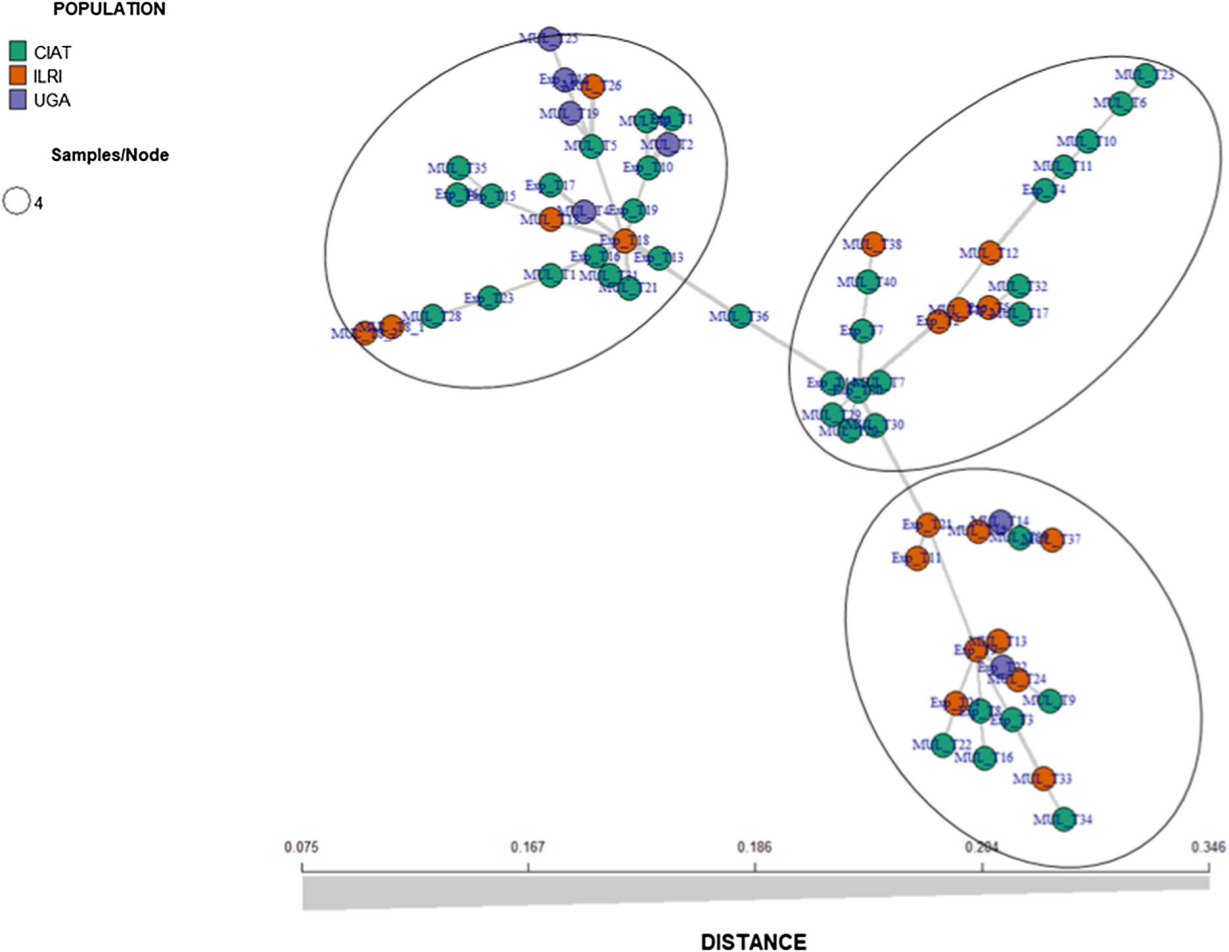
**a** Changes in Delta K with number of subpopulations. **b** Population structure among individuals with K = 3. **c** Population structure among individuals with K = 6

The model-based STRUCTURE, minimum spanning network methods showed the presence of the three divergent groups. The subpopulations within the 65 Lablab accessions were analysed, with the 9,320 polymorphic SNP markers in the STRUCTURE software. The highest peak of delta K was at K = 3 (Fig. 5a), was indicative of three major groups and admixed accessions. However, a second major peak at K = 6 signifies six probable subgroups (Fig. 5b). At a 0.50 membership probability threshold when K = 6, 15 accessions were assigned to Group I, two accessions to Group II, seven accessions to Group III, 14 accessions to Group IV, 12 accessions to Group V, and 15 accessions to Group VI (Fig. 5c). For ΔK = 3, most of the accessions from CIAT, showed the greater probability of ancestral membership for cluster I and III (Table 2).

### Analysis of molecular variance

AMOVA method was employed to analyse lablab populations to deduce the population differentiation using SNP markers. The AMOVA results showed that among diversity explained by 0.57%, and within-cluster diversity explained by 99.43% of the total variation (Table 3).

**Table 3:** Analysis of molecular variance for genetic differentiation among and with clusters of Lablab collection

Source	DF	SS	MS	Est. var	(%)
Among populations	2	5205.06	2602.53	13.47	0.57
Within populations	62	146,618.48	2364.81	2364.81	99.43
Total	64	151,823.54	2372.24	2378.29	100

Genetic differentiation among accession populations (PhiPT) = 0.0056; P = 0.142

*DF* Degree of freedom, *SS* sum of squares, *MS* squares, *Est. var.* estimate of variance, *%* percentage of total variation

P-value is based on 9999 permutations

### Allelic Diversity in the Regional Populations

The allelic diversity in three populations of lablab accessions is presented in Table 2. The number of expected M.L.G. at the smallest sample size ≥ ten based on rarefaction ranged from 7 (UGA) to 10 (CIAT). We detected the highest mean genetic diversity in CIAT population (H = 3.66, G = 39), while the UGA population had the least mean genetic diversity (*H* = 1.95, G = 7). The evenness index was equal to 1 for all accession; hence all occurred at the same frequency. The Nei’s unbiased gene diversity was detected highest in UGA population (Hexp = 0.363) and lowest in ILRI population (Hexp = 0.304). Diversity indices increased with increasing genotypic richness and samples size (Table 2). *H* and G increased linearly as the number of lablab accession (N) increased (Table 2), and this was true for the λ and *H*.

## Discussion

The analysis of a lablab population’s genetic structure is paramount to broaden the knowledge on the genetic base of germplasm for the breeding programs by identifying genetic pools. It enhances the utilisation and conservation of genetic resources. Although many phylogenetic studies have conducted using different markers (Mba and Tohme 2005; Venkatesha et al. 2007), has relied mainly on using low-density molecular markers (Humphry et al. 2002; Konduri et al. 2000; Patil et al. 2009; Sujithra et al. 2009; Wang et al. 2004, 2005). The discovery of genome-wide molecular markers in an organism with restricted genomic data like lablab is possible with genotyping by sequencing approaches, a cost-effective method (Hu et al. 2018; Kilian et al. 2012). This study presents results of the first kind of lablab diversity with advanced molecular marker technologies. We assessed the diversity and population structure in the lablab collection using genome-wide density SNP and SiliconDArT markers (Jaccoud et al. 2001; Kilian et al. 2012). Both SNP and SilcoDart markers used in this analysis resulted in broad agreement albeit varying genomic regions were studied.

Since the Lablab reference genome sequence is in the pipeline of generation, the mungbean genome sequence, was used to map genomic position and distribution of the SNP and SilicoDArT markers. Mungbean (2n = 2x = 22 chromosomes) (Kang et al. 2014) is closely related to lablab (Humphry et al. 2002). indeed, the linkage mapping comparison results showed that mungbean was highly homologous with lablab (Humphry et al. 2002), suggesting that the two species may contrast by an inversion at a particular genomic region. However, both are believed to be all the more phylogenetically far off with the different number of chromosome (11 and 12, respectively) (Humphry et al. 2002). However, a large number of mutations have apparently accumulated after divergence despite their very similar marker orders (Humphry et al. 2002). It was this significant level of homology observed by Humphry et al. (2002) between mungbean and lablab that allowed us to use the mungbean genome as reference. The genome-wide mapping presented the marker distribution and density with most markers located at the peripheral chromosome arms ends, as Kang et al. (2014) reported in mungbean. However, only 7% of the SilicoDArT and 33% of the SNP markers were able to map to the mungbean genome’s seven chromosomes.

Observed clustering implied a wide range of genetic diversity within the *L. purpureus* accessions. Using selected SilicoDart and SNP markers which were distributed across the genome and highly polymorphic makes this study the first of its kind and more robust than earlier reports with low density marker sets like amplified fragment length polymorphism. This study’s results are consistent with previous reports on genetic diversity of collections using agro-morphological, physiological and molecular variables (Basavarajappa and Gowda 2000; Keerthi et al. 2014; Maass 2006; Parmar et al. 2013). This means that the high level of variation among the 65 lablab accessions is attributed to African origin and South America’s collection related to the rich gene pool of the African landraces. In particular, Tefera (2006), showed distinction of the East African landraces from core collection selected to epitomise agro-morphological variation and a wide scope of geographic origins while studying molecular diversity assessment with Amplified fragment length polymorphisms markers. Also, the impact of gene flow and genetic drift on the variation is anticipated to be low as lablab is predominantly self-pollinated.

However, the results are contrary to Venkatesha et al. (2007) who used AFLP markers to study the diversity of 78 *Lablab purpureus* accessions and reported very little genetic diversity within *Lablab purpureus* accessions. In addition, Sultana et al. (2000) reported that 20 landraces studied by randomly amplified polymorphic DNA markers were similar and related to a large portion of the 60 accessions received from Australia than to those of diverse African origins. It seems that labalab in southern Asia is less diverse than that from Africa even though there is impressive agro-morphological variation (Maass et al. 2010).

The clustering of the UGA materials from Uganda, ILRI and CIAT appeared to be based on geographical origin. This is consistent with Venkatesha et al. (2007)’s findings, who reported difference in clustering between southern Indian germplasm collections compared to a set accession from other worldwide germplasm collections that included African accessions. Group 1 consisted more of ILRI and CIAT accessions under SNP clustering but using SilicoDArT markers, the Uganda accessions were evenly distributed in all the 3 groups. This might be due to the type of markers used that is, either dominant markers (SilicoDArT) and co-dominant markers (SNP) (Jaccoud et al. 2001; Kilian et al. 2012).

Analysis of molecular variance showed a high contribution of within-population difference to the total variation implying a high genetic diversity among accessions. This result is substantiated by a low level of genetic variation among the populations, a high pair-wise genetic distance of most accession pairs, and fair representation of accession from all sources in structure analysis clusters (particularly in ΔK = 3). Such difference among the accessions is anticipated due to the self-pollinated reproduction mode in favor of maternal accession regardless of heterozygosity level (Kukade and Tidke 2014; Shrikrishna and Ramesh 2020; Vaijayanthi et al. 2019). The partitioning of molecular variations for the accession population was similar to those reported in previous studies (Humphry et al. 2002; Konduri et al. 2000; Maass et al. 2005; Sujithra et al. 2009; Tefera 2006; Wang et al. 2004). In agreement with the STRUCTURE analysis, NJ tree and minimum spanning analysis showed accession in three distinct groups, but the membership coefficient differed between two analyses. Accessions in group 1, 2 and 3 that clustered exclusive of improved cultivars may require further study to know where they belong, because they could be possessing unique traits of agricultural importance. These observations signify high level of genetic diversity of accessions due to high gene diversity. This is because many Lablab species occur naturally in Africa, a region that represents a center of diversity of the genus (Maass et al. 2005; Maass and Usongo, 2007; Verdcourt 1970).

Our study revealed a high genetic diversity in lablab accessions and their high utility in improvement programs for economic importance traits such as high biomass production, drought tolerance, and pest and diseases resistance. Crosses of distantly related ecotypes could be an excellent strategy to broaden the genetic base. The Lablab genome’s complexity, limited understanding of functional genomics of different genes, and morphological agility within and between the species has limited the pace of Lablab breeding. Therefore, there is a need to enrich the current understanding of Lablab biology and promote the integrated use of conventional and molecular breeding to exploit genetic resources from this collection and those available elsewhere. In addition, characterisation of selected accessions for morphological traits in multiple location may yield superior cultivars for commercial cultivation.

## Conclusions

The genetic diversity and structure of lablab accessions deduced in this study serve as key findings that can be utilised to guide effective management, exploitation, and improvement of accessions to design genetic and marker-trait association studies. The SNP and SilicoDArT markers used in our study, with unification with S.S.R. and SNP markers developed by Konduri et al. (2000), Humphry et al. (2002), Maass et al. (2005), Wang et al. (2004), Tefera (2006) and Sujithra et al. (2009), can serve to heighten the data resources available for lablab improvement using marker assisted selection.

## Supplementary Information

Below is the link to the electronic supplementary material.

Supplementary file1 (DOCX 18 kb)
