# Supplementary material for: Genome-wide diversity and structure variation among lablab [Lablab purpureus (L.) Sweet] accessions and their implication in a Forage breeding program
Source: Genet Resour Crop Evol. 2021 Mar 19;68(7):2997–3010. doi: 10.1007/s10722-021-01171-y (PMC8550355; doi:10.1007/s10722-021-01171-y)
Supplement: Supplementary file 1 — Supplementary file1 (DOCX 18 kb) [file 10722_2021_1171_MOESM1_ESM.docx]

Supplementary Table 1: List of accessions and origin used in this study

| No. | In-house code | Origin | Country | Region |  | No. | In-house code | Origin | Country | Region |
| --- | --- | --- | --- | --- | --- | --- | --- | --- | --- | --- |
| 1 | Exp_T8 | CIAT | Colombia | South America |  | 34 | MUL_T26 | CIAT | Colombia | South America |
| 2 | MUL_T22 | CIAT | Colombia | South America |  | 35 | Exp_T24 | CIAT | Colombia | South America |
| 3 | MUL_T9 | CIAT | Colombia | South America |  | 36 | Exp_T12 | CIAT | Colombia | South America |
| 4 | MUL_T30 | CIAT | Colombia | South America |  | 37 | Exp_T22 | CIAT | Colombia | South America |
| 5 | MUL_T32 | CIAT | Colombia | South America |  | 38 | MUL_T2 | CIAT | Colombia | South America |
| 6 | MUL_T7 | CIAT | Colombia | South America |  | 39 | MUL_T4 | CIAT | Colombia | South America |
| 7 | Exp_T1 | CIAT | Colombia | South America |  | 40 | Exp_T23 | ILRI | Kenya | Africa |
| 8 | Exp_T15 | CIAT | Colombia | South America |  | 41 | Exp_T14 | ILRI | Kenya | Africa |
| 9 | MUL_T11 | CIAT | Colombia | South America |  | 42 | Exp_T16 | ILRI | Kenya | Africa |
| 10 | MUL_T16 | CIAT | Colombia | South America |  | 43 | Exp_T19 | ILRI | Kenya | Africa |
| 11 | MUL_T20 | CIAT | Colombia | South America |  | 44 | MUL_T28 | ILRI | Kenya | Africa |
| 12 | MUL_T23 | CIAT | Colombia | South America |  | 45 | MUL_T1 | ILRI | Kenya | Africa |
| 13 | MUL_T17 | CIAT | Colombia | South America |  | 46 | MUL_T6 | ILRI | Kenya | Africa |
| 14 | Exp_T6 | CIAT | Colombia | South America |  | 47 | Exp_T13 | ILRI | Kenya | Africa |
| 15 | MUL_T29 | CIAT | Colombia | South America |  | 48 | MUL_T21 | ILRI | Kenya | Africa |
| 16 | MUL_T5 | CIAT | Colombia | South America |  | 49 | MUL_T34 | ILRI | Kenya | Africa |
| 17 | MUL_T10 | CIAT | Colombia | South America |  | 50 | MUL_T8_1 | ILRI | Kenya | Africa |
| 18 | Exp_T10 | CIAT | Colombia | South America |  | 51 | Exp_T18 | ILRI | Kenya | Africa |
| 19 | Exp_T7 | CIAT | Colombia | South America |  | 52 | Exp_T11 | ILRI | Kenya | Africa |
| 20 | Exp_T3 | CIAT | Colombia | South America |  | 53 | MUL_T24 | ILRI | Kenya | Africa |
| 21 | MUL_T3 | CIAT | Colombia | South America |  | 54 | MUL_T8_2 | ILRI | Kenya | Africa |
| 22 | Exp_T4 | CIAT | Colombia | South America |  | 55 | MUL_T27 | ILRI | Kenya | Africa |
| 23 | Exp_T17 | CIAT | Colombia | South America |  | 56 | MUL_T19 | ILRI | Kenya | Africa |
| 24 | MUL_T31 | CIAT | Colombia | South America |  | 57 | MUL_T25 | ILRI | Kenya | Africa |
| 25 | Exp_T5 | CIAT | Colombia | South America |  | 58 | MUL_T14 | ILRI | Kenya | Africa |
| 26 | MUL_T18 | CIAT | Colombia | South America |  | 59 | MUL_T35 | UGA | Uganda | Africa |
| 27 | MUL_T33 | CIAT | Colombia | South America |  | 60 | Exp_T20 | UGA | Uganda | Africa |
| 28 | MUL_T15 | CIAT | Colombia | South America |  | 61 | MUL_T40 | UGA | Uganda | Africa |
| 29 | Exp_T9 | CIAT | Colombia | South America |  | 62 | MUL_T36 | UGA | Uganda | Africa |
| 30 | Exp_T21 | CIAT | Colombia | South America |  | 63 | MUL_T39 | UGA | Uganda | Africa |
| 31 | Exp_T2 | CIAT | Colombia | South America |  | 64 | MUL_T37 | UGA | Uganda | Africa |
| 32 | MUL_T12 | CIAT | Colombia | South America |  | 65 | MUL_T38 | UGA | Uganda | Africa |
| 33 | MUL_T13 | CIAT | Colombia | South America |  |  |  |  |  |  |
